# Optimal Timing of Endoscopic Intervention for Acute Variceal Bleeding in Cirrhotic Patients: A Systematic Review and Meta-Analysis

**DOI:** 10.5152/tjg.2025.25432

**Published:** 2025-11-21

**Authors:** Yang Jiang, Yandi Lu, Yang Wang

**Affiliations:** 1 Zhejiang Chinese Medical University School of Medicine, Hangzhou, China; 2 Endoscopy Center, Taizhou Hospital of Zhejiang Province, Zhejiang, China; 3 Department of Gastroenterology, Taizhou Hospital of Zhejiang Province, Linhai, China

**Keywords:** Cirrhosis, endoscopy, gastrointestinal hemorrhage, meta-analysis, mortality, variceal bleeding

## Abstract

**Background/Aims:**

Acute variceal bleeding (AVB) is a severe complication in cirrhotic patients. The optimal timing for endoscopic treatment remains uncertain.

**Materials and Methods:**

A systematic search of PubMed, Embase, and the Cochrane Library was conducted. Outcomes included mortality, rebleeding rate, number of red blood cells (RBC) transfused, length of hospitalization, and other clinical endpoints. Study quality was assessed using the Newcastle–Ottawa Scale (NOS). Meta-analysis was performed using RevMan 5.4.1 (Cochrane, Oxford, United Kingdom) and Stata 18.0 (StataCorp LLC, College Station, United States), with subgroup, sensitivity, and publication bias analyses.

**Results:**

No significant differences were observed between early and delayed endoscopy groups for the outcomes. However, in-hospital mortality was higher in the early endoscopy group (odds ratio (OR) = 1.55, 95% CI: 0.92-2.61, *P* = .10) and reached significance in sensitivity analysis (OR = 1.76, 95% CI: 1.02-3.04, *P* = .04). Additionally, the early group tended to reduce the 6-week bleeding rate (OR = 0.78; 95% CI: 0.49-1.33; *P* = .4) and reached significance in sensitivity analysis (OR = 0.63, 95% CI: 0.43-0.92, *P* = .02). Moreover, the early group tended to require fewer RBC transfusions (mean difference (MD) = −0.62, 95% CI: −1.33 to 0.09, *P* = .09), confirmed in subgroup analysis (MD = −1.32, 95% CI: −2.41 to −0.24, *P* = .02).

**Conclusion:**

No significant differences were found between groups for outcomes. However, subgroup or sensitivity analysis revealed that early endoscopy may increase in-hospital mortality, while reducing 6-week rebleeding and RBC transfusion. Further randomized trials are needed to confirm these findings.

**Main Points**

- Early endoscopic examination may be linked to an elevated in-hospital mortality rate.
- Early endoscopic examination may reduce the 6-week rebleeding rate.
- Early endoscopic examination may mitigate the necessity for red blood cell transfusions.

## Introduction

Acute variceal bleeding (AVB) is the most prevalent cause of acute bleeding in cirrhotic patients and is one of the most severe complications. It is distinguished by a high rate of morbidity, mortality, and recurrence, which poses a direct threat to the lives of patients. Studies have demonstrated that the 1-year incidence of first variceal bleeding in cirrhotic patients is approximately 13%.^1^ Additionally, the 6-week mortality of patients after standardized treatment remains as high as 20% despite the presence of advanced medical treatments.^2^ In this context, the imperative clinical issue of optimizing treatment strategies and means to reduce mortality and recurrence rates has arisen.

The selection and application of therapeutic techniques for gastroscopic therapy are now firmly established in clinical practice; nonetheless, debate persists concerning the timing of the procedure. The American Association for the Study of Liver Diseases advises that endoscopy should be conducted in patients with AVB within 12 hours of admission.^3^ The Baveno VII consensus and the European Society of Gastrointestinal Endoscopy Guideline underscore the necessity of completing endoscopy within 12 hours of presentation subsequent to hemodynamic resuscitation.^4^^,^^5^ The UK guidelines for treating variceal hemorrhage advocate rapid endoscopy upon resuscitation for unstable patients with severe acute upper gastrointestinal bleeding and completion of endoscopy within 24 hours of admission for other bleeding patients.^6^ The Chinese practice guidelines propose that patients with suspected cirrhotic AVB who are hemodynamically stabilized or recovered should undergo gastroscopy within 12-24 hours.^7^ Nonetheless, these proposals primarily rely on expert opinion and lack robust empirical proof.

The timing of endoscopy in patients with AVB has been the subject of relatively few studies to date. Certain studies indicate that the timing of endoscopy does not correlate with death or rebleeding rates in individuals with AVB.^8^^-^^10^ However, 2 studies indicate that mortality in individuals with AVB is independently influenced by the absence of early endoscopy (>12 hours).^11^^,^^12^ The discrepancies across studies have prompted inquiries about the ideal time for endoscopy in individuals with AVB.

This study endeavors to investigate the optimal timing for endoscopic treatment in patients with liver cirrhosis and esophageal variceal hemorrhage by conducting a meta-analysis of the existing literature, in light of the substantial discrepancies in research findings in this field and the absence of a unified consensus.

## Materials and Methods

This study is based solely on secondary analysis of publicly available data from published literature, without direct involvement of human participants, and therefore, does not require ethics committee approval or informed consent.

### Literature Search Strategy

This article was written according to the PRISMA principles. The authors conducted independent searches of PubMed, Embase, and the Cochrane Library from January 1, 1980, to March 31, 2025. The search criteria were as follows: ((liver OR hepatic OR “liver disease” OR “hepatic failure”)) AND ((cirrhosis OR cirrhotic OR “liver cirrhosis” OR “hepatic cirrhosis” OR “portal hypertension”)) AND ((“variceal bleeding” OR “variceal hemorrhage” OR “gastroesophageal varices” OR “esophagogastric varices” OR “acute variceal bleeding” OR “variceal rupture” OR “upper gastrointestinal bleeding” OR “UGIB” OR hematemesis OR melena OR hemorrhage OR bleeding OR AEVB)) AND ((endoscopy OR “endoscopic therapy” OR “endoscopic treatment” OR “endoscopic intervention” OR “urgent endoscopy” OR “emergency endoscopy” OR “EGD” OR “esophagogastroduodenoscopy”)).

### Inclusion and Exclusion Criteria

Observational cohort studies of cirrhotic patients hospitalized for AVB who underwent endoscopic evaluation and whose clinically relevant outcomes were assessed were the inclusion criteria. The following kinds of studies were excluded: (1) Studies that were repetitively published; (2) reviews of the literature, clinical guidelines, meta-analyses, case reports, and conference abstracts; (3) studies involving subjects without liver cirrhosis; (4) studies that did not investigate the timing of endoscopic intervention or did not include relevant data; (5) studies unrelated to patients with AVB; and (6) studies in which datasets were reused or in which key information was unavailable (e.g., the number of patients with cirrhotic AVB, mortality, or rate of rebleeding).

### Data Extraction and Definitions

Researchers retrieved the following information for each included study: first author, year of publication, country, type of study, sample size, use of vasoactive drugs and antibiotics or not, surgical procedure, definition of time of endoscopy, subgroups and number of people at the time of endoscopy, type of bleed studied, rebleeds, deaths, number of red blood cells (RBCs) transfused, hospitalization time, number of successful hemostasis, site of bleeding sites detected, number of intensive care unit (ICU) admissions, number of complications, the number of salvage treatments, and the number of liver transplants. The investigators reached a consensus to resolve their disagreements. Characteristics and Specifications of the Studies Included are shown in Table 1. General information and laboratory tests for patients included in the study are shown in Supplementary Tables 1 and 2.

Rebleeding was characterized by the emergence of hematemesis, black stool, or hematochezia, alongside laboratory findings indicative of hemorrhage (a reduction in hemoglobin exceeding 2 g/dL) or alterations in vital signs (a decline in systolic blood pressure to below 90 mmHg or an elevation in heart rate exceeding 100 beats per minute).^8^^,^^12^ The overall rebleeding summarizes the rebleeding rates at different time points across all studies. The 6-week rebleeding rate is defined as the proportion of patients experiencing bleeding within 6 weeks. The overall mortality rate was calculated by aggregating mortality rates across different time periods in all studies. In-hospital mortality referred to death from any cause occurring during the index hospitalization. The 6-week mortality was defined as all-cause death within 6 weeks following the index bleeding episode or initial endoscopic treatment. The number of RBCs transfused represented the total number of packed RBC units administered during hospitalization or until hemostasis was achieved. Length of hospitalization was defined as the duration (in days) from hospital admission to discharge. The successful hemostasis rate referred to the proportion of patients who achieved initial control of bleeding without the need for rescue therapy within 24 hours after endoscopic treatment. The ICU admission rate was defined as the proportion of patients requiring ICU admission for management or monitoring of complications related to variceal bleeding. The salvage treatment rate encompassed patients who necessitated additional interventions, including balloon tamponade and transjugular intrahepatic portosystemic shunt.^10^ Incidence of complications encompassed adverse events related to the bleeding episode or endoscopic procedure, such as aspiration pneumonia, perforation, hepatic encephalopathy, or infection. The liver transplant rate referred to the proportion of patients who underwent liver transplantation during follow-up after the bleeding episode.

### Evaluation of Quality

The NOS was used to assess the methodological quality of all included non-randomized studies. This scale assesses studies on the basis of 3 domains: the selection of study groups, the comparability of groups, and the determination of either the exposure or outcome. Each study is assigned a maximum of 9 points by the NOS. The quality evaluation was performed by 2 separate evaluators, and any inconsistencies were addressed by discussion or contact with a third reviewer. High-quality studies were defined as those scoring ≥7, moderate-quality studies as those scoring 4-6, and low-quality studies as those scoring ≤3. Three of the studies that were included were of moderate quality, while the remaining studies were of high quality (Table 2).

### Statistical Analysis

RevMan 5.4.1 (Cochrane, Oxford, United Kingdom) and Stata 18.0 software (StataCorp LLC, College Station, United States) were employed to conduct this meta-analysis. Continuous variables were analyzed using the mean and standard deviation (SD) or the median and interquartile interval. Percentages were employed to characterize categorical variables. Categorical variables are expressed as percentages, while continuous variables are represented as means and SD. Continuous outcomes were expressed as mean differences (MDs) and 95% CIs, while dichotomous outcomes were expressed as odds ratios (ORs). Heterogeneity was evaluated using *I*^2^ statistics. A fixed-effects model (Mantel–Haenszel method) was employed to calculate the pooled estimates if there was no statistically significant heterogeneity between studies (*I*^2^ < 50%, *P* > .1). Otherwise, a random-effects model (DerSimonian and Laird method) was employed. If the *P* value was less than .05, statistical significance was assessed. Subgroup or sensitivity analyses are implemented to mitigate substantial heterogeneity. The publication bias is assessed using Egger’s test, and a *P* value of less than .05 indicates a substantial publication bias.

## Results

### Literature Selection

**Figure 1.**
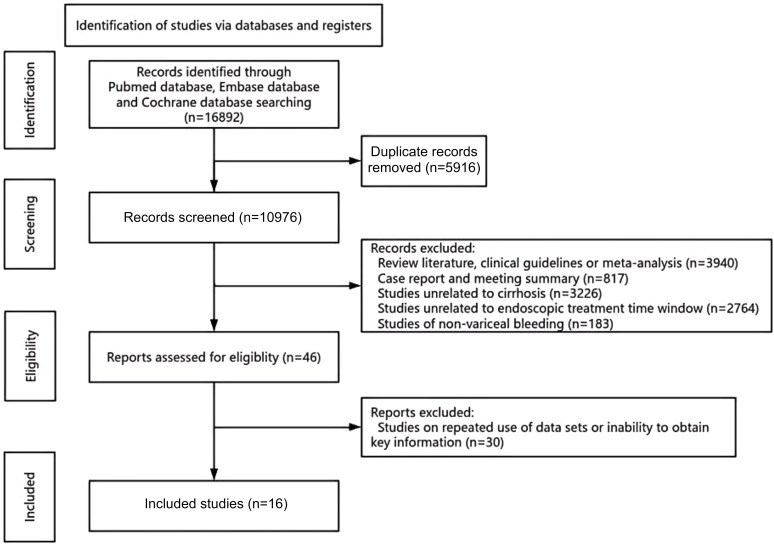
PRISMA flowchart.

The flowchart of study selection for inclusion in the meta-analysis is illustrated in Figure 1. The literature search yielded a total of 16 892 (3983, 12 480, 429) studies. Articles that were duplicated (n = 5916) were excluded. Review literature, clinical guidelines, and meta-analyses (n = 3940); case reports and meeting summaries (n = 817) were excluded following an initial screening of study categories. These were followed by studies unrelated to cirrhosis (n = 3226), studies unrelated to the endoscopic treatment time window (n = 2764), studies of non-variceal bleeding (n = 183), and finally studies on repeated use of data sets or inability to obtain key information (n = 30). Ultimately, 16 studies remained.

### Primary Outcomes

**Figure 2.**
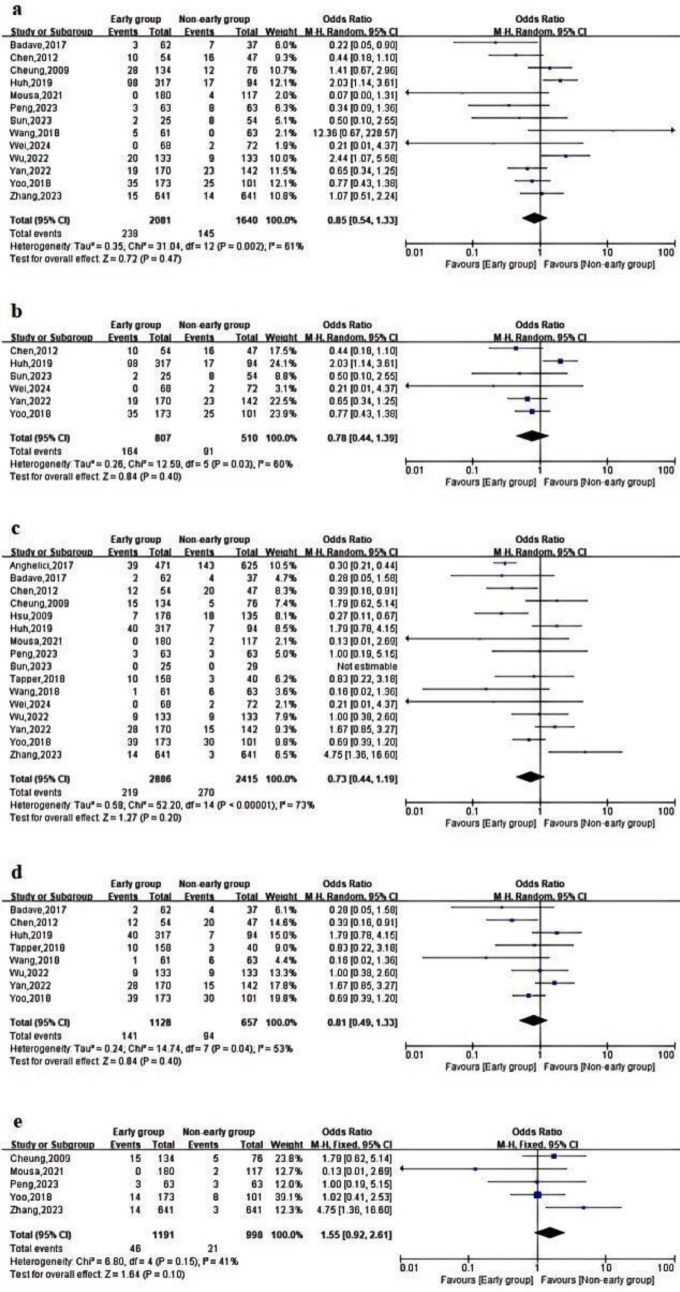
Forest plot comparing the primary metrics of early and delayed endoscopy groups. (a) Overall rebleeding rate; (b) 6-week rebleeding rate; (c) overall mortality; (d) 6-week mortality; (e) in-hospital mortality.

Table 3 displays the primary outcomes of the studies that were incorporated. Regarding the overall rebleeding rate, 13 studies indicated no statistically significant difference between the early endoscopy group and the delayed endoscopy group (OR = 0.85; 95% CI = 0.54-1.33; *P* = .47, *I*^2^ = 61%) (Figure 2A). A meta-analysis of 6 studies revealed no significant difference in 6-week rebleeding rates between early and delayed endoscopy^8^^-^^10^^,^^12^^-^^18^^,^^20^ (OR = 0.78; 95% CI = 0.44-1.39; *P* = .4, *I*^2^ = 60%) (Figure 2B).

Regarding mortality rates, an analysis of overall mortality across 17 studies revealed no statistically significant differences (OR = 0.73; 95% CI = 0.44-1.19; *P* = .2, *I*^2^ = 73%) (Figure 2C). For in-hospital mortality, all included studies used a 12-hour cutoff point, and there was no significant statistical difference between early endoscopy and delayed endoscopy^8^^,^^10^^,^^12^^,^^16^^,^^17^ (OR = 1.55; 95% CI = 0.92-2.61; *P* = .10, *I*^2^ = 41%) (Figure 2E). A meta-analysis of 8 studies showed no significant difference in 6-week mortality between early and delayed endoscopy^9^^,^^15^^,^^16^^,^^18^^-^^20^ (OR = 0.81; 95% CI = 0.49-1.33; *P* = .4, *I*^2^ = 53%) (Figure 2D).

### Secondary Outcomes

**Figure 3.**
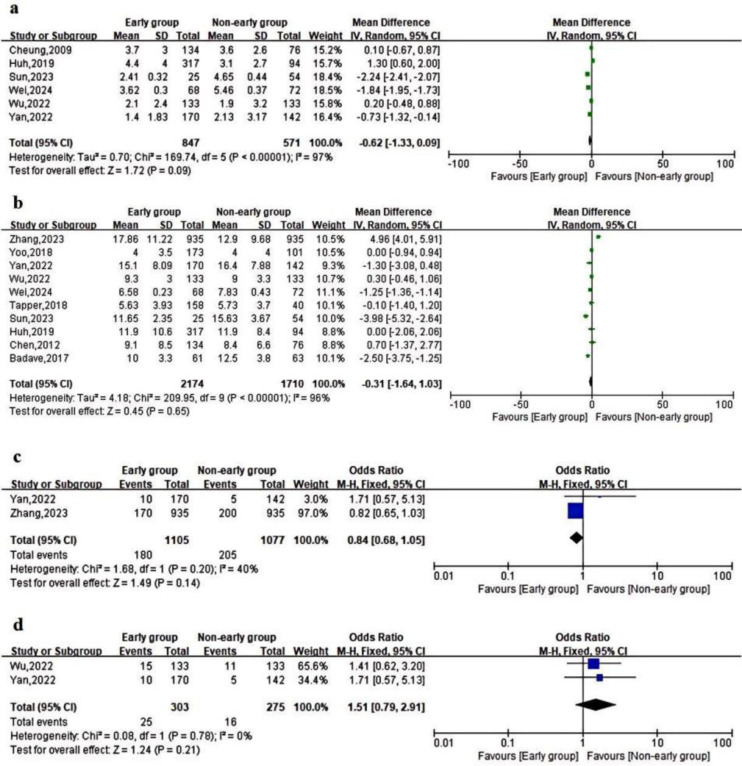
Forest plot comparing the secondary metrics of the Early Endoscopy Unit and the delayed Endoscopy Unit. (a) Number of red blood cells transfused; (b) length of hospitalization; (c) ICU admission rate; (d) salvage treatment rate.

Supplementary Table 3 displays the secondary outcomes of the studies that were incorporated. A meta-analysis of data from 6 studies determined that the number of RBCs transfused does not exhibit a significant difference between early and delayed endoscopy^9^^,^^11^^-^^15^^,^^20^ (MD = −0.62; 95% CI = −1.33 to 0.09; *P* = .09, *I*^2^ = 97%) (Figure 3A).

The length of hospitalization was not significantly different between early endoscopy and delayed endoscopy, as demonstrated by a meta-analysis of 9 study results^8^^,^^9^^,^^12^^-^^16^^,^^19^^,^^20^ (MD = −0.31; 95% CI = −1.64 to 1.03; *P* = .65, *I*^2^ = 96%) (Figure 3B).

There were no statistically significant differences between the included studies in terms of ICU admission rate and rescue treatment rate after the meta-analysis (Figure 3C and D).

### Sensitivity Analysis

To evaluate the robustness of the pooled estimates and to identify potential sources of heterogeneity, sensitivity analyses were performed for the overall population and subgroups using a stepwise exclusion approach. These analyses involved the sequential omission of individual studies, particularly those differing in key clinical or methodological characteristics—such as sample size, bleeding etiology (esophageal vs. gastric varices), and timing of endoscopy. The results demonstrated that pooled outcomes for overall mortality, overall rebleeding rate, 6-week rebleeding rate, number of RBC units transfused, and length of hospitalization were relatively stable, suggesting that no single study disproportionately influenced these estimates. However, the results for in-hospital mortality, 6-week mortality, and 6-week rebleeding rate were less stable. In the examination of in-hospital mortality, the exclusion of the paper by Mousa et al^17^ markedly diminished heterogeneity, perhaps due to the study’s exclusive focus on bleeding following esophageal variceal rupture^15^ (OR = 1.76; 95% CI = 1.02-3.04; *P* = .04, *I*^2^ = 29%). Excluding the research by Chen et al,^18^ the analysis of 6-week mortality diminished heterogeneity, potentially attributable to the limited sample sizes of the investigation^16^ (OR = 1.11; 95% CI = 0.72-1.71; *P* = .63, *I*^2^ = 30%). Similarly, excluding the study by Huh et al^20^ significantly reduced the heterogeneity of the 6-week rebleeding rate results (OR = 0.63, 95% CI = 0.43-0.92, *P* = .02, *I*^2^ = 0%). This reduction may be attributable to the faster mean heart rate observed in patients undergoing early endoscopy in this study, potentially indicating more severe disease and suggesting potential selection bias.

### Subgroup Analysis

A subgroup analysis was performed according to the time interval between hospital admission or the onset of the first bleeding episode and endoscopic treatment, categorizing the study population into 2 groups using 6-hour and 12-hour thresholds, respectively. There was no statistically significant difference between the 2 groupings in terms of rebleeding, mortality, or length of hospitalization. Patients who underwent endoscopy within 6 hours required substantially fewer RBC transfusions, according to a meta-analysis of the 2 studies with a 6-hour cut time (MD = −1.32, 95% CI = −2.41 to −0.24, *P* = .02, *I*^2^ = 92%) (Supplementary Figures 1 and 2).

### Publication Bias

Publication bias was assessed for overall rebleeding rate, overall mortality, and length of hospitalization. Visual inspection of the funnel plots revealed no apparent asymmetry, and Egger’s regression test did not indicate significant publication bias for overall rebleeding (*P* = .529) or overall mortality (*P* = .327) (Supplementary Figure 3). However, significant publication bias was detected for length of hospitalization (*P* = .002). For other outcomes, formal assessment of publication bias was not conducted due to the inclusion of fewer than 10 studies, in accordance with PRISMA guidelines.

## Discussion

A previous meta-analysis by Bai et al^24^ also investigated the optimal time window for treating bleeding from esophageal and gastric varices in cirrhotic patients. Their findings showed a significantly lower overall mortality rate in the early endoscopy group compared to the delayed endoscopy group, which differs from the results of the present study. Compared to Bai et al’s study, this research has the following advantages: first, it included more studies (16 vs. 9); second, more refined subgroup analyses were conducted, including not only a 12-hour subgroup analysis but also a 6-hour subgroup analysis; finally, sensitivity analyses were performed by sequentially excluding individual studies to assess the robustness of the findings.

This meta-analysis included 16 publications that examined the correlation between the time of endoscopy and clinical outcomes in AVB. Only one of these papers was a Western study and did not specifically contend for the time window. In contrast, a search was conducted for studies on the time window for non-variceal hemorrhage, but only a substantial number of Western studies were identified. A review of the literature revealed the following potential reasons.^25^^,^^26^ One reason is that the etiology of cirrhosis in Western countries is predominantly influenced by alcoholic liver disease, which is distinguished by the delayed onset and late manifestation of portal hypertension. Hepatitis B virus is the most prevalent disease in Eastern countries and is recognized for its ability to induce portal hypertension and esophageal variceal bleeding (EVB) at an earlier stage. The “12-hour rule” is a “quality indicator” in specific Western hospitals, which also adhere to more standardized processes and ethics. The “12-hour rule” is the principal focus of “quality indicators” in specific Western hospitals. As a result, the global research on the selection of endoscopic therapeutic apertures for patients with ruptured esophagogastric varices and cirrhosis hemorrhage is restricted, and the research has a greater clinical urgency and research value.

The meta-analysis revealed no statistically significant differences in any outcome indicators between early endoscopy and delayed endoscopy. In spite of this, additional discoveries were made during the subsequent investigation. Initially, the combined effect of in-hospital mortality indicates that early endoscopy may be associated with a higher mortality rate. Although this difference did not reach statistical significance (*P* = .1), it may have potential clinical relevance. The difference achieved statistical significance after 1 study was excluded in a sensitivity analysis. This may be due to the fact that early endoscopy did not provide sufficient blood resuscitation, which increases the risk of complications such as cardiopulmonary failure due to hemodynamic instability in patients.^5^ Additionally, bleeding may have impaired endoscopic visualization, which increases the likelihood of errors and misdiagnosis. Moreover, some studies failed to perform Propensity Score Matching (PSM) or Inverse Probability Weighting (IPW) on patient data, leading to a higher likelihood of including critically ill patients in the early endoscopy group. These patients may be more prone to in-hospital mortality. Secondly, patients who underwent early endoscopy may have lower 6-week mortality rates and require fewer RBC transfusions compared to those who underwent delayed endoscopy, as these outcomes achieved statistical significance in sensitivity analyses or subgroup analyses. These may be due to the fact that early endoscopy can directly locate and stop bleeding during the active bleeding phase, while simultaneously reducing venous pressure and controlling subsequent rebleeding, thereby reducing 6-week rebleeding rate and the need for transfusions.^6^

Studies have shown that the Child classification of patients with ruptured varices influences postoperative outcomes following endoscopic treatment.^27^^,^^28^ It was hypothesized that the optimal endoscopic treatment time window may also vary among patients with cirrhosis and variceal bleeding classified into different Child grades. In each of the studies that comprised the analysis, risk stratification (Child or Model for End-Stage Liver Disease (MELD) grading) was performed for all patients; however, only 3 studies conducted subgroup analyses to determine the optimal endoscopic treatment time window for patients with different risk grades. In contrast to those who underwent endoscopic examinations after 6 hours, Zhang et al^8^ demonstrated that the 5-day treatment failure rate for Child-B patients was substantially higher in those who underwent examinations within 6 hours. Peng et al,^10^ in a subgroup analysis of Child-B/C patients, found that although the results were not statistically significant, both the 5-day treatment failure rate and in-hospital mortality rate were higher in the <12-hour group compared to the >12-hour group.^14^ This may be due to the fact that, for patients with suboptimal baseline conditions, it may be more crucial to optimize clinical status through early fluid resuscitation than to perform endoscopic hemostasis prematurely. There was no statistically significant difference in outcomes between patients with Child-C grade and MELD scores >17 in 2 studies conducted by Zhang. This may be attributable to the tiny sample size, and additional research is required to verify this discovery.

One limitation of this study is that all included studies were retrospective cohort studies with multiple biases, and most did not apply PSM or IPW. Consequently, there were differences in the severity of illness between patients undergoing early endoscopy and those undergoing delayed endoscopy. Clinically, physicians tend to prioritize endoscopy for patients whose condition is deteriorating. Therefore, those who underwent early endoscopy may have been in worse condition. To further validate the impact of different endoscopy time periods on the prognosis of cirrhotic patients with ruptured esophagogastric fundal varices hemorrhage, more prospective randomized controlled studies are necessary. A prospective randomized controlled study is currently in progress.^29^ This randomized controlled trial is stratified by the Child-Pugh grades and clinical presentation. Participants are randomly assigned to the early endoscopy examination and the delayed endoscopy groups within each stratum in a 1 : 1 ratio. This arrangement aids in the verification of between-groups differences. The true impact of endoscopy timing on short-term prognosis was evaluated under various liver function statuses and clinical acute manifestations on the basis of baseline balance. The stratified design of this study is of exceptional scientific quality and is anticipated to address the current issue of confounding bias, which is founded on retrospective evidence.

In addition to the above point, this study also has the following limitations. Initially, the included studies exhibited substantial discrepancies in the definition of the endoscopy time window’s starting point. While some studies designated the time of the first bloody vomiting or the appearance of black stool as the starting point, others designated the time of the last bleeding event or the patient’s admission to the hospital as the starting point. Peng et al^10^ conducted a subgroup analysis using varying time definitions, and the results of the 2 groups were inconsistent. Consequently, the interpretation of the final combined effect may be influenced by the inconsistent endoscopic examination duration definitions across studies. Secondly, different studies employ varying definitions for “early” or “delayed” endoscopy. While some heterogeneity was addressed through subgroup analyses, certain discrepancies remain unresolved. For instance, the definition of delayed endoscopy varies across studies: some set the threshold at >12 hours, while others use 12–24 hours or 12–48 hours. Moreover, the endoscopic treatment procedures exhibited a high degree of variability among the studies. Some studies exclusively employed endoscopic variceal ligation, while others employed a combination of tissue adhesive injections, sclerotherapy, or alternated between procedures. This inconsistency in intervention may have had a heterogeneous impact on patient prognosis. In addition, there was inconsistency in the types of variceal bleeding among patients included in the studies: some studies exclusively enrolled patients with EVB, while others included both esophageal and gastric variceal bleeding patients. Esophageal variceal bleeding typically presents with large volumes and rapid bleeding rates, readily leading to shock and acute hemodynamic instability. In contrast, gastric variceal bleeding tends to be slower in both volume and rate, and may be difficult to detect in the early stages. Consequently, this inconsistency in bleeding type may result in significant differences in patient outcomes, potentially contributing to heterogeneity in the pooled analysis. Besides, the evidence base was predominantly derived from Asian cohorts; among the 16 included studies, only one was conducted in a Western setting. This predominance of Asian studies introduces a potential regional bias, as differences in healthcare infrastructure, patient demographics, and diagnostic and treatment standards across various Asian countries may limit the generalizability of these findings to other regions, particularly Western countries. Lastly, this study displayed a high degree of statistical heterogeneity in several outcome measures, and factors such as hemorrhage severity and liver function status could potentially contribute to this heterogeneity. Although subgroup and sensitivity analyses partially accounted for the heterogeneity, residual bias should not be disregarded and should be further confirmed by prospective studies with a standardized design and high quality.

## Supplementary Materials

Supplementary Material

## Figures and Tables

**Table 1.** Characteristics and Specifications of the Studies Included

First Author (Year)	Country	Research Type	Adjustment Method	Use of Vasoactive Drugs and Antibiotics	Method of Endoscopic Treatment	Definition of Endoscopy Time	Endoscopic Examination Time Group (Number of Patients)	Bleeding Patterns
Zhang ^8^	China	RCS	PSM	Yes	NA	A→E	<6 hours and 6-24 hours (935), <12 hours and 12-24 hours (935)	AGVB
Sun ^13^	China	RCS	None	Yes	EVL or EIS or EVO	F→E	< 12 hours (25),12-48 hours (29), >48 hours (25) and failure of medication (23)	AGVB and PHGH
Yan ^9^	China	RCS	None	Yes	EVL and/or EIS and/or EVO	A→E	<6 hours (170) and 6-24 hours (142)	AGVB
Wei ^14^	China	RCS	None	Yes	EIS and EVO	F→E	<6 hours (68), 6-24 hours (72) and expectant treatment (67)	AGVB
Wu ^15^	China	RCS	PSM	Yes	EVL and/or EIS and/or EVO	A→E	≤12 hours (133) and >12 hours (133)	AGVB
Yoo ^16^	Korea	RCS	IPW	Yes	EVL/EVL and EVO	A→E	<12 hours (173) and ≥12 hours (101)	AGVB
Peng ^10^	China	RCS	PSM	Yes	EVL and/or EIS and/or EVO	L→E/A→E	<12 hours (63) and ≥12 hours (63), <24 hours (155) and ≥24 hours (155), <48 hours(199) and ≥48 hours (199)	AGVB
Mousa ^17^	Egypt	RCS	None	Yes	EVL	A→E	<12 hours (180) and 12-24 hours (117)	AEVB
Chen ^18^	China (Taiwan)	RCS	None	Yes	EVL	A→E	6-12 hours (54) and >12 hours (47)	AEVB
Tapper ^19^	USA	RCS	None	Yes	EVL and/or EIS	A→E	<12 hours (158) and 12-24 hours (40)	AEVB
Huh ^20^	Korea	RCS	None	Yes	EVL and/or EIS	A→E	<12 hours (317) and 12-24 hours (94)	AGVB
Hsu ^11^	China (Taiwan)	RCS	None	Yes	EVL and/or EVO	NA	<15 hours (176), ≥15 hours (135)	AGVB
Cheung ^12^	China (Taiwan)	RCS	None	Yes	EVL and/or EVO	NA	≤4 hours (57) and >4 hours (153), ≤8 hours (102) and >8 hours (108), ≤12 hours (134) and >12 hours (76)	AGVB
Wang ^21^	China	RCS	None	NA	EVL	NA	12-24 hours (61), >24 hours (63)	AGVB
Anghelici ^22^	Moldova	RCS	None	NA	EVL and/or EVO	F→E	<12 hours (471) and ≥12 hours (625)	AGVB
Badave ^23^	India	RCS	None	NA	NA	NA	<6 hours (62) and 6-24 hours (37)	AGVB

A→E, the time interval from admission to the beginning of endoscopy; F→E, the time interval from the first bleeding to the beginning of endoscopy; L→E, the time from the last bleeding to the start of endoscopy; AEVB, acute esophageal variceal bleeding; AGVB, acute gastroesophageal variceal bleeding; EIS, endoscopic injection sclerotherapy; EVL, endoscopic variceal ligation; EVO, endoscopic variceal obturation; IPW, inverse probability weighting; NA, not available; PHGH, portal hypertensive gastropathy hemorrhage; PSM, propensity score matching; RCS, retrospective cohort study.

**Table 2.** Methodological Qualitative Assessment (Newcastle–Ottawa Scale)

First Author (Year)	Selection	Comparability	Outcome	Total
Zhang ^8^	4	2	2	8
Sun ^13^	4	2	3	9
Yan ^9^	4	1	3	8
Wei ^14^	4	2	2	8
Wu ^15^	4	2	3	9
Yoo ^16^	4	2	3	9
Peng ^10^	4	1	2	7
Mousa ^17^	3	1	3	7
Chen ^18^	4	2	3	9
Tapper ^19^	3	2	2	7
Huh ^20^	3	2	2	7
Hsu ^11^	3	1	1	5
Cheung ^12^	4	2	3	9
Wang ^21^	3	2	3	8
Anghelici ^22^ Abstract	3	0	1	4
Badave ^23^ Abstract	3	0	1	4

**Table 3.** Primary Outcomes

First Author (Year)	Number of Rebleeding	Death Toll
Zhang^8^	5-day rebleeding: <6 hours/6-24 hours: 28/27,12 hours/12-24 hours: 15/14	In-hospital: <6 hours/6-24 hours: 18/11, <12 hours/12-24 hours: 14/3
Sun ^13^	≤6 weeks: <12 hours/12-48 hours: 2/4, >6 weeks: <12 hours/12-48 hours: 4/5	6-month: <12 hours/12-48 hours: 0/0
Yan ^9^	6-week: <6 hours/6-24 hours: 19/23	6-week: <6 hours/6-24 hours: 28/15
Wei ^14^	6-week: <6 hours/6-24 hours: 0/2	Death from illness: <6 hours/6-24 hours: 0/2
Wu ^15^	30-day: ≤12 hours/>12 hours: 25/23	30-day: ≤12 hours/>12 hours:9/9
Yoo ^16^	6-week: <12 hours/≥12 hours: 35/25	In-hospital: <12 hours/≥12 hours: 14/8,6-week: <12 hours/≥12 hours: 39/30
Peng ^10^	5-day: <12 hours/≥12 hours: 3/8	In-hospital: <12 hours/≥12 hours: 3/3
Mousa ^17^	<12 hours/12-24 hours: 0/4	In-hospital death: <12 hours/12-24 hours: 0/2
Chen ^18^	6-week: 6-12 hours/>12 hours: 10/16	6-week: 6-12 hours/>12 hours: 12/20
Tapper ^19^	NA	6-week: <12 hours/12-24 hours: 10/3
Huh ^20^	6-week: <12 hours/12-24 hours: 98/17	6-week: <12 hours/12-24 hours: 40/7
Hsu ^11^	NA	In-hospital: <15 hours/≥15 hours: 7/18
Cheung ^12^	≤12 hours/>12 hours: 28/12	In-hospital: ≤12 hours/>12 hours: 15/5
Wang ^21^	72 hours-2 weeks: 12-24 hours/>24 hours: 5/0, >2 months:12-24 hours/>24 hours: 19/21	6-week death: 12-24 hours/>24 hours: 1/6
Anghelici ^22^	NA	<12 hours/>12 hours: 39/143
Badave ^23^	30-day: <6 hours/6-24 hours: 3/7	30-day: <6 hours/6-24 hours: 2/4

NA, not available.

## Data Availability

The data that support the findings of this study are available on request from the corresponding author.
