# Supplementary material for: Optimal Timing of Endoscopic Intervention for Acute Variceal Bleeding in Cirrhotic Patients: A Systematic Review and Meta-Analysis
Source: Turk J Gastroenterol. 2025 Nov 21;37(3):281–91. doi: 10.5152/tjg.2025.25432 (PMC12994423; doi:10.5152/tjg.2025.25432)
Supplement: Supplementary Material [file supplementary_material.pdf]

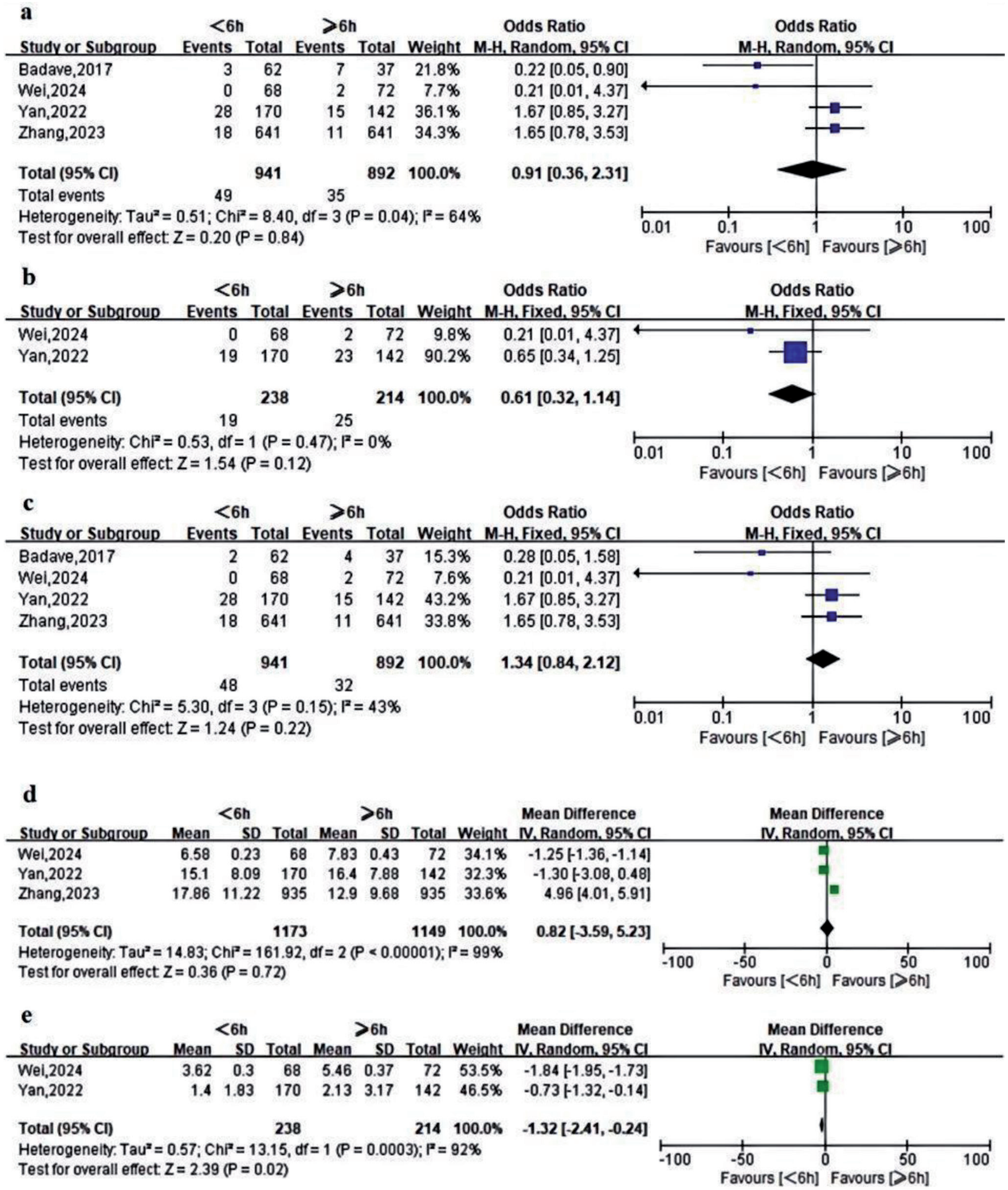

Supplementary Figure 1. Forest plot of outcome indicators between the less than 6 hours group and the more than 6 hours group. (a) Overall rebleeding rate; (b) 6-week rebleeding rate; (c) overall mortality; (d) length of hospitalization; (e) number of red blood cells transfused.

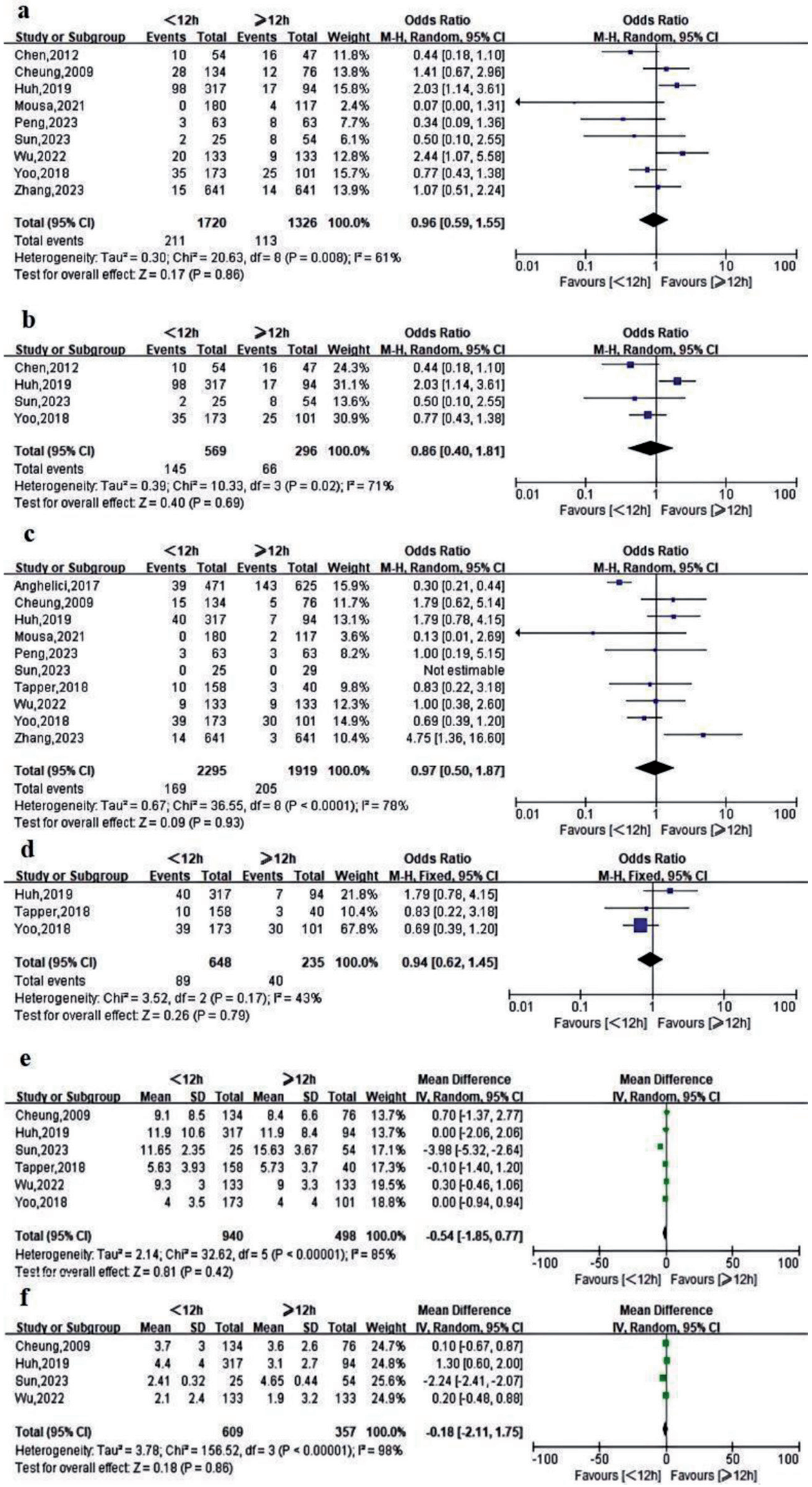

Supplementary Figure 2. Meta-analysis of outcome indicators between the less than 12-hour group and the more than 6-hour group. (a) Overall rebleeding rate; (b) 6-week rebleeding rate; (c) overall mortality; (d) 6-week mortality; (e)length of hospitalization; (f) number of red blood cells transfused.

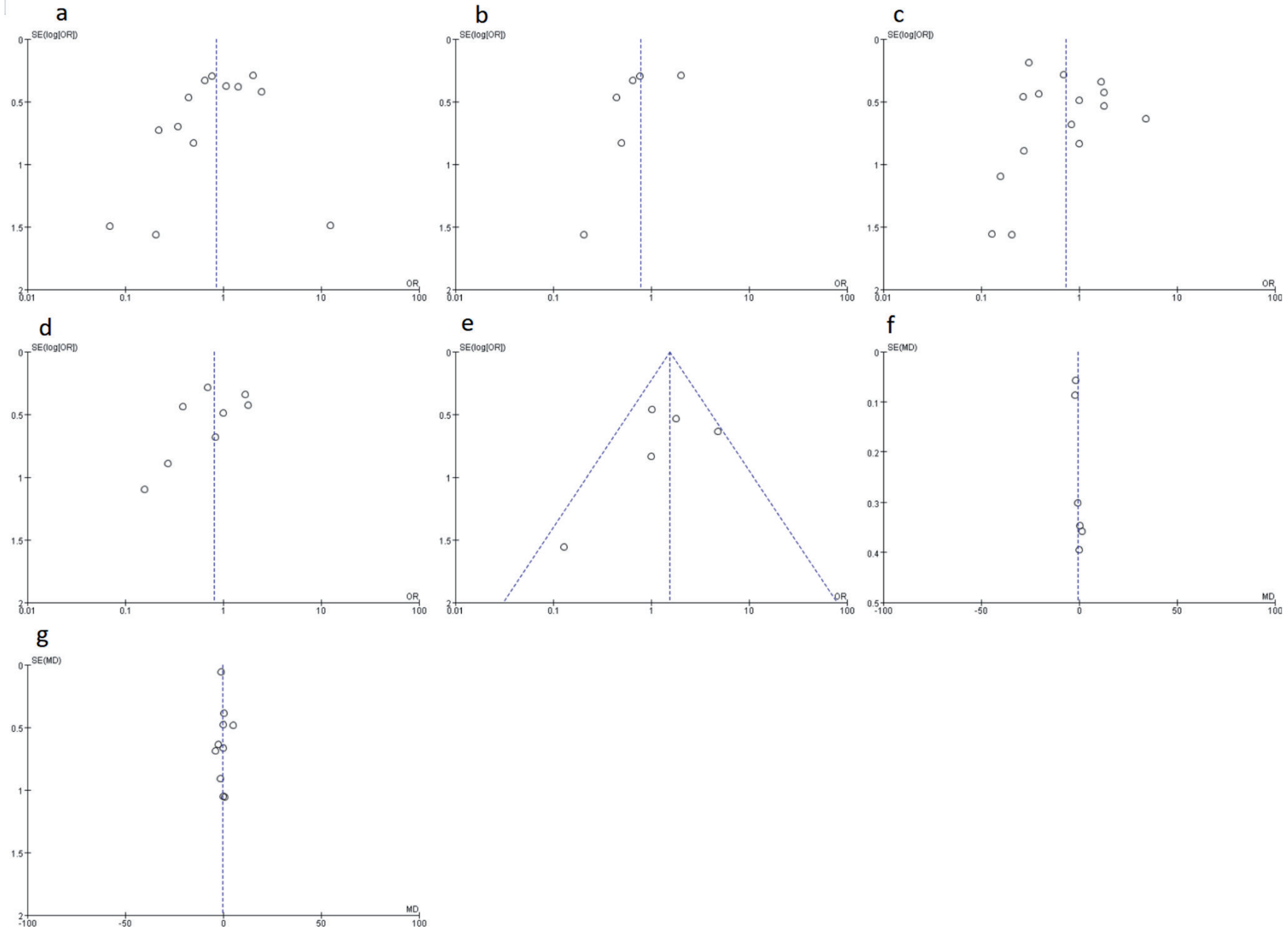

Supplementary Figure 3. Funnel plot for assessment of heterogeneity of included studies. (a) Overall rebleeding rate; (b) 6-week rebleeding rate; (c) overall mortality; (d) 6-week mortality; (e) in-hospital mortality; (f) number of red blood cells transfused; (g) length of hospitalization.

Supplementary Table 1. General data of the patients

First author (year)	Etiology of cirrhosis	Child-Pugh score	MELD score	With a bleeding history	Previous medication use
Zhang [8]	NA	A:857,B:1844,C:618	NA	<6h/6-24h: 495/495	NA
Sun [13]	Viral:65,alcoholic:11,primary biliary: 3,cryptogenic:19,others:4	A:23,B:46,C:33	<12h/12-48h11.65±2.58/11.08±2.68*	NA	NA
Yan [9]	HBV:225,HCV:12,AIH:29,Alcohor:19,Other:39	8.05± 1.69*	14.21± 4.99*	NA	Clopidogreluse/Aspirinuse/ Betablockers:18/5/23
Wei [14]	NA	A:26,B:73,C:41	NA	NA	NA
Wu [15]	HBV:187,Alcohol:22,HCV:4,Schistosome:9,HBV:11,Biliary:10,Other:33	≤12h/> 12h:7.4±1.6/7.4±1.7*	≤12h/>12h:10.1±3.6/9.9±3.8*	NA	NA
Yoo [16]	HBV:136,HCV:25,Alcohol:68,Others:43	9.1 ± 3.5*	15.9± 7.8*	<12h/≥12h: 2/6	NA
Peng [10]	HBV/HCV:222/47	A:165,B+C:333	11.92±4.61*	NA	NA
Mousa [17]	HBV:280,HCV:12NASH:5	A:24,B:64,C:209	NA	NA	NA
Chen [18]	Viral hepatitis/Alcohol/ Others:74/18/9	9(7.5-11)#	13(10-20)#	NA	NA
Tapper [19]	Alcohol:107,HCV:46,HBV:6,NAFLD:17,Cryptogenic:8,Other:13,Multiple:42	NA	14(11-21)#	NA	NA
Huh [20]	Alcohol:240,HBV:108,HCV:28,Other:6	8.3±2.4*	12.1±6.9*	NA	NA
Hsu [11]	Alcohol:46,HBV:150,HCV:78	NA	11.6(8.5-14.8)#	NA	NA
Cheung [12]	Alcohol:139,HCV:59,Cryptogenic:20,Other:27	NA	14.3±5.3*	NA	NA
Wang [21]	NA	A:31,B+C:93	<17:26 98,≥17:98	NA	NA
Anghelici [22]	NA	NA	NA	NA	NA
Badave [23]	NA	NA	NA	NA	NA

MELD, Model for End-Stage Liver Disease. NA, not available.*Mean ± SD. #Median [interquartile range (IQR) 25th–75th percentile].

Supplementary Table 2. Laboratory examination of patients

First author (year)	PLT(109/L)	HB(g/L)	Serum albumin(g/L)	TB(umol/L)	ALT(U/L)
Zhang [8]	75.00 (62.25)#	76.00 (30.00)#	NA	NA	25.00 (23.00)#
Sun [13]	NA	NA	NA	NA	NA
Yan [9]	100.21±55.70*	76.43±21.10*	28.48±5.62*	38.01±24.85*	49.40±72.47*
Wei [14]	NA	NA	NA	NA	NA
Wu [15]	≤12h/> 12h:83.9±51.5/83.3±49.6*	≤12h/> 12h:84.2±22.6./82.3±22.0*	≤12h/> 12h:32.4±5.3/32.4±5.7*	≤12h/> 12h:27.4±18.9/26.4±17.3*	NA
Yoo [16]	117±79*	NA	NA	NA	NA
Peng [10]	87.44±58.82*	77.53±22.51*	30.57±6.53*	25.01±18.35*	NA
Mousa [17]	<12h/12-24h:83±23.8/82±28.6*	<12h/12-24h:79±26/78±23*	<12h/12-24h:29±0.9/27±0.2*	<12h/12-24h:14.1±6.2/12.4±6.2*	<12h/12-24h: 62±17.2/61±21.1*
Chen [18]	111(67.5-160.5)#	NA	NA	32.49(20.52-59.85)#	44.5 (28-80.8)#
Tapper[19]	106±74*	9.7(8.1-11.5)#	NA	NA	NA
Huh [20]	102.3± 60.9*	NA	NA	NA	NA
Hsu [11]	NA	NA	NA	NA	NA
Cheung [12]	117±91*	90±20*	NA	56±76*	NA
Wang [21]	NA	68.2±18.6*	NA	55.5±25.7*	62.0±24.0*
Anghelici [22]	NA	NA	NA	NA	NA
Badave [23]	NA	NA	NA	NA	NA

PLT, Platelet counts; HB, Haemoglobin; TB, Total bilirubin; ALT, Alanine Aminotransferase; NA, not available.
*Mean ± SD. #Median [interquartile range (IQR) 25th–75th percentile].

Supplementary Table 3. Secondary outcomes

First author (year)	Number of red blood cells transfused	Hospitalization time(h)	Number of successful hemostasis	Number of icu occupancy	Number of complications	Number of rescue treatments	Number of liver transplants
Zhang [8]	NA	<6h/6-24h:17.86±11.22/12.9±9.68*	NA	<6h/6-24h:170/200	NA	NA	NA
Sun [13]	<12h/12-48h:2.41±0.32/4.65±0.44*	<12h/12-48h:11.65±2.35/15.63±3.67*	NA	NA	<12h/12-48h:0/1	NA	NA
Yan [9]	<6h/6-24h:1.40±1.83/2.13±3.17*	<6h/6-24h:15.10±8.09/16.40±7.88*	NA	<6h/6-24h:10/5	NA	<6h/6-24h:10/5	NA
Wei [14]	<6h/6-24h:3.62±0.30/5.46±0.37*	<6h/6-24h:6.58±0.23/7.83±0.34*	NA	NA	NA	NA	NA
Wu [15]	≤12h/>12h:2.1±/2.4/1.9±3.2*	≤12h/>12h:9.3±3.0/9.0±3.3*	NA	NA	NA	≤12h/>12h:15/11	NA
Yoo [16]	NA	<12h/≥12h:4.0±3.5/4.0±4.0*	NA	NA	NA	NA	<12h/≥12h:14/11
Peng [10]	NA	NA	NA	NA	NA	NA	NA
Mousa [17]	NA	NA	NA	NA	NA	NA	NA
Chen [18]	NA	NA	NA	NA	NA	NA	NA
Tapper [19]	NA	<12h/12-24h:5.63±3.93/5.73±3.70*	NA	NA	NA	NA	NA
Huh [20]	<12h/12-24h:4.4±4.0/3.1±2.7*	NA	NA	NA	NA	NA	NA
Hsu [11]	NA	NA	NA	NA	NA	NA	NA
Cheung [12]	≤12h/>12h:3.7±3.0/3.6±2.6*	≤12h/>12h:9.1±8.5/8.4±6.6*	≤12h/12h:129/74	NA	NA	NA	NA
Wang [21]	NA	12-24h/>24h:10.0±3.3/12.5±3.8*	NA	NA	NA	NA	NA
Anghelici [22]	NA	NA	NA	NA	NA	NA	NA
Badave [23]	NA	NA	NA	NA	NA	NA	NA

NA, not available.*Mean ± SD.
